# 3D-Printed Polycaprolactone/Hydroxyapatite Bionic Scaffold for Bone Regeneration

**DOI:** 10.3390/polym17070858

**Published:** 2025-03-23

**Authors:** Feng-Ze Wang, Shuo Liu, Min Gao, Yao Yu, Wen-Bo Zhang, Hui Li, Xin Peng

**Affiliations:** 1 Department of Oral and Maxillofacial Surgery, Peking University School and Hospital of Stomatology and National Center for Stomatology and National Clinical Research Center for Oral Diseases and National Engineering Research Center of Oral Biomaterials and Digital Medical Devices and Beijing Key Laboratory of Digital Stomatology and NHC Key Laboratory of Digital Stomatology and NMPA Key Laboratory for Dental Materials, No.22, Zhongguancun South Avenue, Haidian District, Beijing 100081, China; fengzewang@bjmu.edu.cn (F.-Z.W.); liushuo1001@yeah.net (S.L.); tcyuyao@126.com (Y.Y.); michaelzhang1016@126.com (W.-B.Z.); 2 Department of VIP Dental Service, Department of Geriatric Dentistry, Peking University School and Hospital of Stomatology, Beijing 100081, China; dzgaomin@163.com; 3 School of Systems Science and Institute of Nonequilibrium Systems, Beijing Normal University, Beijing 100875, China; huili@bnu.edu.cn

**Keywords:** polycaprolactone (PCL), hydroxyapatite (HA), three-dimensional printing, gyroid, bionic scaffold, bone formation

## Abstract

The limitations of traditional, autologous bone grafts, such as the scarcity of donor material and the risks of secondary surgical trauma, have spurred the development of alternatives for the repair of large bone defects. Bionic bone scaffolds fabricated via fused deposition modeling (FDM)—a three-dimensional (3D) printing technique—are considered promising. While gyroid-structured scaffolds mimic the complex micro-architecture of cancellous bone, their application in FDM 3D printing remains understudied. Furthermore, no consensus has been reached on the ideal pore size for gyroid scaffolds, which is influenced by the infill density. In this study, we fabricated five groups of polycaprolactone/hydroxyapatite (PCL/HA) scaffolds with different infill densities (40%, 45%, 50%, 55%, and 60%) using a solvent-free filament preparation method. Scanning electron microscopy (SEM) observation showed that all scaffolds exhibit an interconnected porous structure. The scaffold with the 55% infill density, featuring a pore size of 465 ± 63 μm, demonstrated optimal hydrophilicity and mechanical properties comparable to natural cancellous bone. In addition, this scaffold supported cellular bridging within its pores and showed the highest alkaline phosphatase (ALP) activity and calcium salt deposition. Our findings offer novel insights into the design of gyroid-like scaffolds and their fabrication via FDM, paving the way for potential clinical applications.

## 1. Introduction

Bone defects caused by traumas, infections, tumors, and osteonecrosis are frequently encountered in clinical practice, and these seriously affect the life of patients and impose a heavy economic burden on society. Although autologous bone grafting has been the golden standard for repairing bone defects, limited autologous bone has prompted the development of new alternative strategies based on bone tissue engineering [1].

With the development of three-dimensional (3D) printing technology, complicated porous scaffolds that mimic natural bone can be manufactured. Research on bone scaffolds is now focusing on bio-inspired design and dynamic function regulation [2]. The bionic structure is essential to maintain the self-renewal and pluripotent uniqueness of stem cells [3]. The gyroid structure is very similar to the structure of the trabecular bone [4]. Inspired by the micro-structure of the butterfly wing, a gyroid-like scaffold was developed, and it displayed excellent mechanical properties and an interconnecting porous structure that promote nutrient flow and osteogenic differentiation of bone mesenchymal stem cells (BMSCs) [5,6,7]. Compared with scaffolds with grid or raster-angle structures, gyroid-like scaffolds had more concave surfaces that are more suitable for cell adhesion, proliferation, and spreading. The 3D-printing techniques that are commonly used to fabricate gyroid-like scaffolds are stereolithography (SLA), digital light processing (DLP), and selective laser melting (SLM) [7,8,9]. Among them, SLA and DLP printers use photosensitive resins for printing and require chemical solvents for post-processing of the printed samples, which were prone to cytotoxicity [10]. SLM printers use bio-inert metals, but the compressive modulus of the printed object was higher than that of natural bone, leading to stress shielding. Fused deposition modeling (FDM) is one of the most widely used technologies due to its wide choice of materials, low cost, high printing accuracy, and bio-compatibility [11]. The use of FDM for the fabrication of suitable scaffolds to repair bone defects is very promising. However, the use of FDM to manufacture the gyroid structure is challenging because the gyroid structure is not always continuous along the cross-section, which makes path planning difficult compared with SLA, DLP, and SLM [4]. Few studies have attempted to manufacture gyroid-like scaffolds by using FDM, and further research is needed to optimize the involved printing parameters [12].

Polymer and ceramic composites are preferable when using FDM technology for bone tissue engineering owing to their low price, wide range of sources, and excellent bio-compatibility [13]. Among the biocompatible materials, polycaprolactone (PCL) and hydroxyapatite (HA) have attracted considerable attention and have been approved by the U.S. Food and Drug Administration (FDA) for bone tissue engineering. PCL has excellent bone regeneration potential, and its low melting point makes it an ideal material for 3D-printing technology [14]. Ecologically derived biopolymers (e.g., PLA and chitosan) provide sustainability benefits; they face challenges such as brittleness, rapid degradation, inadequate mechanical stability for structural applications, and higher production/purification costs [15]. HA, similar to the inorganic constituent of natural bone, exhibits not only intrinsic osteoinductivity but also potent osteoimmunomodulatory capabilities [16]. However, optimizing the parameters of the FDM implant is infrequently conducted using PCL/HA as raw material [17,18]. In our previous study, we have obtained a scaffold with an interconnected structure after optimizing the printing parameters of an FDM printer and found that the addition of HA (20% weight ratio) to PCL scaffolds dramatically improved mechanical properties, making them similar to the properties of bone trabeculae [19]. In the FDM printer, the filament is melted using a temperature-controlled print head, and then, the digitally designed model is printed on a print plate via layer-by-layer deposition [11]. A previous paper has reported the manufacturing of PCL/HA composite filaments by the solvent method [20]. However, solvent residues may have an adverse effect on the cell growth.

The pore size of the scaffold is an important factor that affects the mechanical properties, nutrient transport, metabolic waste removal, and osteogenic differentiation of MSCs [21,22,23]. The minimum pore size recommended in the literature to promote osteointegration and avoid pore-clogging during 3D printing is >300 μm [24,25,26]. A smaller pore size (<300 μm) induces the chondrogenesis of BMSCs, while a larger pore size (>900 μm) reduces the cell attachment area and the extent of intercellular bridging in the scaffold [27]. Current research on selecting the optimal pore size predominantly centers on Ti6Al4V alloy scaffolds with grid or diamond-shaped lattice designs [28,29,30]. However, no consensus has been reached on the optimal pore size for bone formation based on a PCL/HA gyroid-like scaffold. Moreover, the relationship between bone regeneration and pore size still requires in-depth study. In the design of bionic scaffold, a simple way to adjust the pore size is to adjust the infill density, thereby also affecting the mechanical properties [31]. Few studies have investigated the impact of the infill density on the mechanical and osteogenic properties of FDM implants. Therefore, the optimal pore size obtained by adjusting the infill density to promote osteogenesis must be determined.

In this work, we developed a process that ensured uniform mixing of 20% HA in PCL, thus avoiding stress concentrations caused by uneven mixing, and this uniform mixing was verified using scanning electron microscopy (SEM) images of longitudinal sections of the PCL/HA composite filament. We have fabricated five types of scaffolds with different infill densities (40%, 45%, 50%, 55%, and 60%) by using the prepared PCL/HA filament. Further, to determine the optimal infill density of the bionic gyroid scaffolds, the hydrophilicity, mechanical properties, and bio-compatibility were assessed by using water contact angle test, live/dead cell staining and the cell count kit 8 (CCK8) assay, and by performing bone formation experiments, including alkaline phosphatase (ALP) activity and Alizarin Red Staining.

Our study systematically examined the relationship between optimizing the infill density of FDM-printed PCL/HA gyroid scaffolds and their hydrophilicity, mechanical properties, cellular bridging-induced mechanostimulation, and osteogenic performance.

## 2. Materials and Methods

### 2.1. PCL and PCL/HA Filament Fabrication

PCL (average Mn = 45,000, ρ = 1.146 g/mL, and product No: P871873) and hydroxylapatite (HA, particle size = 200 nm, purity = 94.5–100%, and product No: H875580) were purchased from Shanghai Macklin Biochemical Technology Company (Shanghai, China). Pure PCL powder was put into the hopper of a filament extruder (3DPANY, Zhengzhou, China) to fabricate the PCL filament. The steps for fabricating the PCL/HA composite filament were as follows: Firstly, the PCL powder was melted in a water bath. Then, the HA (the ratio of weights of HA to PCL was 20:80) was added and stirred thoroughly. Next, the PCL/HA mixture in its melted state was added to the hopper of the filament extruder to form the filament via extrusion. The filament extruder operated with two temperature gradients: an intermediate temperature near the feeding hopper and an extrusion temperature at the nozzle. The extruded filament was directed through a water-cooling channel to ensure uniform solidification. The diameter was continuously monitored using a laser gauge. Once stabilized at 1.75 ± 0.05 mm, which was considered the functional diameter for FDM printing, for 10 min, the filament was collected by a winding machine to produce PCL and PCL/HA composite filaments. The functional PCL and PCL/HA filaments were dried in a vacuum dryer and stored within Ziploc bags.

### 2.2. Characterization and Mechanical Testing of PCL and PCL/HA Filaments

Images and elemental distribution of the cross-section of the PCL and PCL/HA filaments were obtained using SEM (SU8010, Hitachi, Tokyo, Japan) and energy-dispersive X-ray spectroscopy (EDX), respectively. In addition, the tensile properties of the two filaments were evaluated using a universal testing machine (MARK 10, New York, NY, USA).

### 2.3. Bionic Scaffolds Fabrication and Characterization

#### 2.3.1. Bionic Scaffolds Fabrication

The scaffolds were designed using Tinkercad (Tinkercad, Autodesk, San Francisco, CA, USA) and PrusaSlicer (PrusaSlicer, Version 2.3.1, Prague, Czech Republic) software. The pore size of the scaffolds was adjusted by setting different infill densities. The content of the G-code file included the layer height (200 μm), infill pattern (gyroid), and infill density (40%, 45%, 50%, 55%, and 60%). The scaffolds were printed using an FDM printer (PRUSA MK3S, Prague, Czech Republic), and the printer’s nozzle diameter was 400 μm. First, the filaments were loaded into the 3D printer, and then, the extrusion temperature, extrusion speed, heat bed temperature, and nozzle extrusion volume were adjusted, as detailed in our previously published paper [19]. All the scaffolds were dried in a vacuum drier (BKMAM, Changsha, China) and stored within Ziploc bags.

#### 2.3.2. Characterization of Bionic Scaffolds

Images of the surface and cross-sectional morphology of the PCL and PCL/HA scaffolds were obtained using SEM. EDX was performed to confirm the elemental composition on the surface of the scaffolds. The pore size of the bionic scaffolds was measured via SEM imaging, with 10 pores randomly selected per scaffold (*n* = 3).

Raman spectroscopy was performed with a 633 nm laser as the excitation light source, to evaluate the functional groups in the PCL powder, HA particles, PCL scaffold, and PCL/HA scaffold in the wavenumber range of 500–3500 cm^−1^ (Renishaw InVia Reflex spectrometer, Renishaw, Wotton-under-Edge, UK). To analyze the phase composition of the PCL powder, HA particles, PCL scaffold, and PCL/HA scaffold, X-ray diffraction (XRD; D8 ADVANCE, Bruker, Switzerland) analysis was performed with Cu Kα X-ray (wavelength λ = 1.5406 Å) radiation at a scanning rate of 10°/minute and in the 2θ range of 10–80°.

### 2.4. Hydrophilicity of Scaffolds

A drop-shape analyzer (Optical Tensiometer, Theta, Biolin, Sweden) was used to measure the contact angle, i.e., the angle formed by a liquid at the three-phase boundary point where a solid, liquid, and gas intersect. The contact angle is an important parameter to evaluate the hydrophilicity of the scaffolds. The scaffolds were cleaned in an ultrasonic bath for 10 min. Then, after 3 µL of deionized water was dropped onto the surface of the scaffolds, the contact angles at 0 s, 1 s, 3 s, and 5 s were recorded automatically. The average of the left and right contact angles at 5 s was calculated for each sample, and the samples of each group is 3 (*n* = 3).

### 2.5. Mechanical Testing of Scaffolds

The compressive strength of the scaffolds (15 × 15 × 4 mm^3^, *n* = 3) was evaluated using a microcomputer-controlled electronic universal testing machine (CARE Measurement & Control, China) at a cross-head speed of 1 mm/min. The compressive strength and strain were calculated as follows:

α = applied load (N)/compressed area of the surface (mm^2^).

β = H/H0

where α, β, H, and H0 are the compressive strength (MPa), compressive strain, deformation of the specimen (mm), and original height of the specimen (mm). The stress–strain curves were generated using Origin 2017 software (OriginLab Corporation, Northampton, MA, USA).

The compressive modulus of the specimen was calculated based on the two points in the linear interval of the stress–strain curve by using the following formula:

E = (α2 − α1)/(β2 − β1)

The elastic modulus in this study was calculated at 2% strain of the bionic scaffolds.

### 2.6. Thermogravimetric Analysis

To determine the amount of HA in the PCL/HA scaffold, thermogravimetric analysis (TGA) was conducted using STA 2500 with an Al_2_O_3_ pan (Netzsch, Germany). The main principle was to place the samples (PCL powder, HA particles, PCL scaffold, and PCL/HA scaffold) in the Al_2_O_3_ pan independently. The samples were heated to 500 °C at a rate of 10 °C/minute. The weight loss was recorded using a high-sensitivity balance.

### 2.7. Cell Culture

Mouse bone marrow stromal cells (BMSCs) were purchased from American Type Culture Collection (ATCC, Manassas, VA, USA). The cells were cultured in an incubator at 37 °C with 5% CO_2_ using alpha-minimum eagle’s medium (α-MEM) supplemented with 10% (*v*/*v*) fetal bovine serum (FBS) and 1% (*v*/*v*) penicillin-streptomycin. The medium was changed every two days. Cells were detached using 0.25% trypsin when the confluence reached 80–85%. The scaffolds were sterilized with 75% ethanol for 1 h followed by drying under sterile conditions at room temperature for 1 h, and then, the scaffolds were further sterilized under UV light for 1 h. Next, the cells were seeded in the 24-well plate (200 μL cell suspension + 300 μL fresh medium) to conduct the in vitro experiments, following the manufacturers’ instructions.

### 2.8. Bio-Compatibility of Scaffolds

#### 2.8.1. CCK8 Test

Scaffolds co-cultured with BMSCs in each group were transferred into a new 24-well plate at days 2, 4, and 14. Then, a mixture of 500 μL of fresh medium and 50 μL of CCK-8 reagent (Beyotime, Shanghai, China) was added into each well and incubated at 37 °C in an incubator for 1 h. Next, 100 μL of the solution from each well was added into the 96-well plate, and the 450 nm absorbance values were recorded using an ELISA reader (EL×808, BioTek, Winooski, VT, USA) to evaluate the cell viability of different groups.

#### 2.8.2. Live-Dead Cell Staining

After days 4 and 14, scaffolds with BMSCs were washed with the PBS buffer twice and transferred into a new 24-well plate for live/dead cell staining using the Calcein-AM/ propidium iodide (PI) staining kit (Beyotime, China). Calcein-AM and PI reagents were added to the detection buffer at a volume ratio of 1:1000 to prepare the staining solution. Then, 250 μL of the staining solution was added into each well to cover the scaffolds, followed by incubation at 37 °C in an incubator for 0.5 h. At the end of the incubation, live and dead cells were observed under an inverted fluorescence microscope (Olympus, Japan), and the images were merged using the ImageJ software (version 1.54g, National Institutes of Health, Rockville Pike Bethesda, MD, USA).

#### 2.8.3. Cell–Scaffold Adhesion

After 4 and 14 days of co-culture, the medium was aspirated from each well, and the scaffolds were washed with the PBS buffer three times. Then, 500 μL of 4% paraformaldehyde was added into each well to fix cells for 2 h at 4 °C. The scaffold–cell complexes were dehydrated in a series of gradient alcohols (30%, 50%, 70%, 80%, 90%, 95%, and 100%), and the scaffold was dehydrated twice at each alcohol gradient, each time for 15 min. The dehydrated samples were dried and sprayed with gold to observe the growth of BMSCs on the scaffold surface (SEM, SU8010, Hitachi, Japan).

### 2.9. Alkaline Phosphatase (ALP) Activity

An ALP activity assay was used to assess the scaffolds in promoting early osteogenic differentiation in BMSCs. Briefly, after the scaffolds were co-cultured with BMSCs for 2 days, the medium was changed to an osteogenic induction medium (α-MEM + 10% (*v*/*v*) FBS + 1% (*v*/*v*) penicillin-streptomycin + 50 μg/mL L -ascorbic acid + 10mM β-Glycerophosphate + 10 nM dexamethasone). After 7 and 14 days of co-culture, the scaffolds were washed using PBS twice and transferred into a new 24-well plate. Then, 1 mL of 0.1% Triton X-100 was added into each well to lyse the cells for 1 h at 4 °C. The lysate was centrifuged at 2500 rpm for 20 min at 4 °C. The supernatant was collected to detect ALP activity by using an alkaline phosphatase assay kit (Beyotime, China) and by following the manufacturer instructions. Meanwhile, ALP staining was performed on days 7 and 14 with the BCIP/NBT alkaline phosphatase color development kit (Beyotime, China).

### 2.10. Alizarin Red S (ARS) Staining

During the differentiation of stem cells into osteoblasts, calcium salts were deposited on the cell surface, forming calcium nodules. After 2 days of co-culture with BMSCs in a growth medium, the scaffolds were transferred into a new 24-well plate, and the osteogenic induction medium was added. After 21 days, the scaffolds were washed using PBS twice, and the cells were fixed using 4% paraformaldehyde (Beyotime, China) for 30 min at room temperature. The calcium nodules were stained using the Alizarin Red S staining kit (Beyotime, China) for 30 min at room temperature. The scaffolds were rinsed using distilled water three times thoroughly. Images of staining were recorded by using a stereomicroscope (Zeiss, Oberkochen, Germany). The ratio of the area of calcium salt deposition on the scaffold surface was calculated by the “threshold color function” in the ImageJ software. For quantitative analysis, 10% chlorohexadecyl pyridine (Solarbio, China) was used to dissolve calcium nodules at room temperature for 30 min, and the absorbance was measured at 570 nm using an ELISA reader (EL×808, BioTek, United States).

### 2.11. Statistical Analysis

Data were expressed as the mean ± standard deviation (Mean ± SD). Data were analyzed with the Student’s t-test and one-way analysis of variance (ANOVA) with post-hoc analysis to compare the difference between groups. Significance levels were considered to be *p* < 0.05 (*). All statistical analyses were performed using SPSS 26.0 (IBM, Armonk, NY, USA).

## 3. Results and Discussion

### 3.1. FDM Printing Bionic Scaffolds with PCL/HA Filament

**Figure 1:**
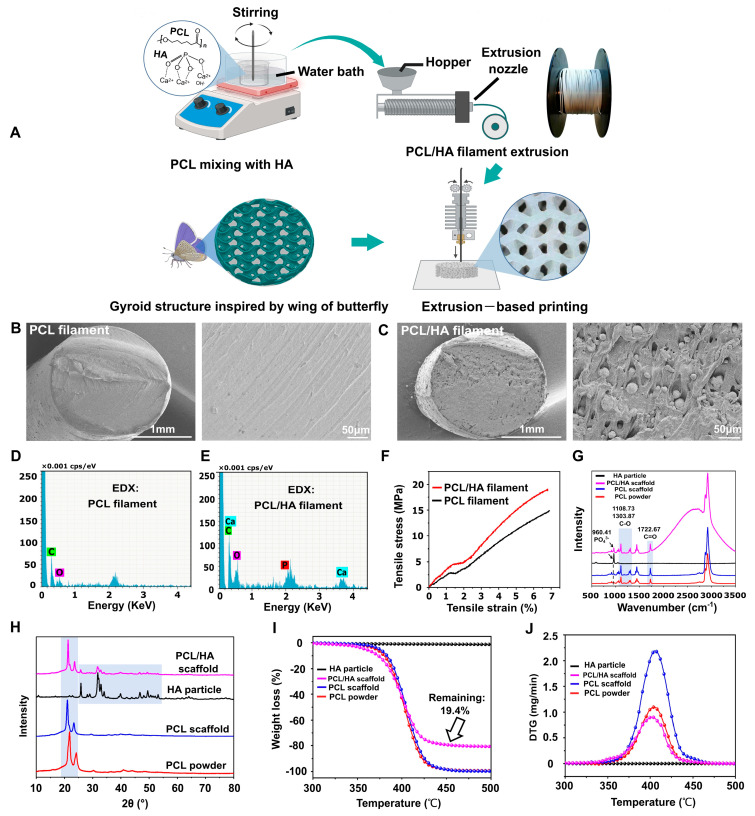
Filaments fabrication and bionic scaffold printing. (**A**) Schematic diagram of filament fabrication and bionic scaffold printing. Created with BioRender.com. (**B**,**C**) SEM images of cross-sections of PCL and PCL/HA filaments. (**D**,**E**) Elemental analysis of cross-sections of PCL and PCL/HA filaments. (**F**) Tensile stress–strain curves of PCL and PCL/HA filaments. (**G**) Raman spectra analysis of HA particles, PCL/HA scaffold, PCL scaffold, and PCL powders. (**H**) XRD patterns of HA particles, PCL/HA scaffold, PCL scaffold, and PCL powders. (**I**,**J**) TGA and DTG results for HA particles, PCL/HA scaffold, PCL scaffold, and PCL powders.

PCL and PCL/HA composite filaments were fabricated by using the 3DPANY machine. The manufacturing parameters were as follows: PCL filament: intermediate temperature: 65 °C, extrusion temperature: 60 °C, and screw speed: 2 RPM and PCL/HA filament: intermediate temperature: 160 °C, extrusion temperature: 130 °C, and screw speed: 7 RPM. After drying in a vacuum oven at 30 °C for 4 h, the PCL and PCL/HA composite filaments were prepared for subsequent use. Then, inspired by the microstructure of a butterfly wing [5], a gyroid-like scaffold was developed and fabricated using an FDM printer (Figure 1A).

The cross-sectional morphology of the PCL filament was smooth, and HA was distributed in the PCL/HA filament (Figure 1B,C). The EDX results showed the presence of elements C and O in the PCL filament and C, O, Ca, and P elements in the PCL/HA filament (Figure 1D,E). In addition, the results of the mechanical test showed that the tensile properties of the PCL/HA composite filament were substantially better than those of the PCL filament (Figure 1F).

The detailed parameters of scaffold printing were as follows: layer height (200 μm); infill pattern (gyroid); infill density (40%, 45%, 50%, 55%, and 60%); printing speed: 80 mm/s; table temperature: 30 °C; and production time: 3 min for a scaffold with a volume of 15 × 15 × 2 mm^3^. The Raman spectra showed that the PCL/HA scaffold and HA particles had the same PO_4_^3−^ group at a wavenumber of 960.41 cm^−1^, which was in accordance with current research [32], demonstrating that the HA was successfully anchored in the PCL (Figure 1G). Further, the spectra showed C=O and C-O stretching at 1722.67, 1108.73, and 1303.87 cm^−1^, which were characteristic of PCL, reflecting the main carbon skeleton of the scaffolds and consistent with existing studies [32,33] (Figure 1G). Figure 1H showed the XRD patterns of four groups: PCL particles, PCL scaffold, PCL/HA scaffold, and nHA powder. The PCL-based materials (particles, PCL scaffold, and PCL/HA scaffold) exhibited peaks at 2θ = 20–25°, confirming the crystalline PCL phase as reported [34]. In contrast, the nHA powder and PCL/HA scaffold displayed characteristic HA peaks at 2θ = 25–30°, 30–40°, and 46–54°, consistent with known HA crystallinity [35,36]. Notably, no HA-related peaks were detected in the PCL particle or PCL scaffold groups. Hence, XRD spectra of the PCL/HA scaffolds confirmed the presence of both HA and PCL phases. The PCL and HA phases in the PCL/HA scaffolds were consistent with the PCL and HA particles, indicating that the crystalline phases of PCL and HA did not change during filament fabrication and 3D printing (Figure 1H).

The results of TGA and DTG experiments showed that no significant weight changes were seen in the HA particle, and the remaining three groups (PCL powder, PCL scaffold, and PCL/HA scaffold) all showed a decrease in weight from around 350 °C, indicating the vaporization of the PCL. TGA analysis revealed that the PCL/HA scaffold exhibited 10% and 50% mass loss at 357 °C and 400 °C, respectively. Notably, the PCL + 20% HA scaffold had a remaining weight of 19.4% HA after 350–500 °C, representing the successful preparation of well-mixed PCL + 20% HA composite filaments (Section 2.1) for 3D printing (Figure 1I). We firmly believed that this workflow will provide a reference for the manufacture of polymer–ceramic composite filaments. The DTG curve showed that the PCL powder, the PCL/HA scaffold, and the PCL scaffold all exhibited the highest rate of weight reduction around 400 °C (Figure 1J); this indicates that the thermodynamic properties of PCL remain unchanged during filament fabrication and 3D printing.

**Figure 2:**
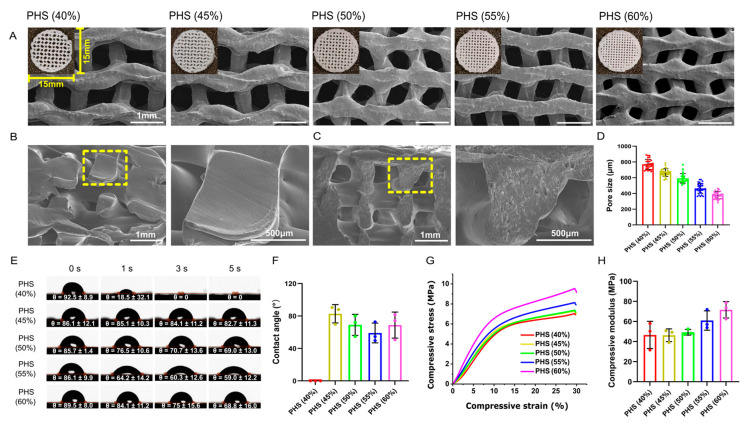
Morphology, hydrophilicity, and mechanical testing results for bionic scaffolds. (**A**) General and SEM images of five groups of scaffolds. (**B**,**C**) Cross-sectional view of PCL and PCL/HA scaffold. (**D**) SEM image showing pore sizes of scaffolds with different infill densities of 40%, 45%, 50%, 55%, and 60%. (**E**,**F**) Water contact angle test of scaffolds (*n* = 3), with PHS (55% infill) showing the smallest water contact angle. (**G**) Stress–strain curves of scaffolds. (**H**) Compressive modulus of scaffolds (*n* = 3).

The distribution of HA particles on the surface of the PCL/HA scaffold was evident, and all the scaffolds had interconnected porous microstructures (Figure 2A–C). The scaffold pore size decreased gradually as the infill density increased (Figure 2D). The internal porous structure is a key factor that determines the scaffold’s performance. An appropriate pore structure, size, and inter-connectivity facilitates the transportation of nutrients and metabolic waste as well as the formation of blood vessels and new bone.

### 3.2. Optimal Hydrophilicity of PHS (55% Infill) with Mechanical Strength Similar to Cancellous Bone

Surface hydrophilicity is a significant parameter that affects the adhesion of cells on the scaffold and can be assessed on the basis of the contact angle [37]. The contact angle of PCL/HA scaffolds with an infill density of 40% was 0 at 3 s, which may be related to the excessive pore size that leads to the rapid penetration of water droplets. In the remaining four groups, the contact angle on the surface gradually decreased with the increase in the infill density from 45% to 55%. However, by PHS (60% infill), the contact angle increased again, which may be related to the pore size being too small and the resultant, highly dense filling affecting the liquid penetration. PHS (55%) had the smallest contact angle, exhibiting the highest hydrophilicity (Figure 2E,F). This may be because, when the infill structure inside the scaffold was consistent, as the pore size decreased, the mean curvature of the structure in the scaffold increased, which was more favorable for liquid penetration [38,39].

The appropriate scaffold mechanical properties needed to improve implantation success and promote early implantation osseointegration needed to be determined [40,41]. Each scaffold group underwent compressive testing with stress/strain calculations (*n* = 3). Due to highly similar stress–strain curves across triplicate samples within each group, one dataset per group was selected for Origin plotting to visualize mechanical performance differences among 40–60% infill densities (Figure 2G). The stress–strain curves showed that, when the infill density was increased from 40% to 60%, the compressive strength increased from 0 MPa to about 10 MPa, and the stress yield inflection point was reached at 30% compressive strain (Figure 2G). When the infill density was increased from 40% to 60%, the compressive modulus gradually increased from 46.6 MPa to 71.6 MPa (Figure 2H), which is within the range for cancellous bone (compressive modulus: 10–1570 MPa), thus indicating clinical application potential [42,43]. These mechanical properties ensured compatibility with the physiological environment, unlike stiffer materials such as polyetheretherketone (PEEK), which has an elastic modulus of 3–4 GPa [44]. PEEK, a bioinert polymer, demonstrates high compressive strength and modulus compared to PCL and ecologically derived biopolymers but lacks inherent biodegradability and osteoinductivity [45]. Although PCL exhibited lower ultimate strength compared to PEEK, its gradual degradation ensured a dynamic transition of mechanical loads to newly formed bone and avoided long-term stress shielding, mimicking natural remodeling processes.

The elastic modulus of cancellous bone varied significantly across anatomical sites (e.g., femur, tibia, femoral neck, and craniofacial regions) [46]. Scaffold design must therefore align with site-specific mechanical demands. Our scaffold targeted low-load applications (e.g., craniofacial defects), where excessive stiffness could cause stress shielding. Its modulus matched non-weight-bearing trabecular bone, balancing early stability (preventing collapse) with gradual load transfer to regenerating tissue, mimicking natural remodeling. For high-load scenarios (e.g., femoral head and neck, femur, and tibia), to better emulate the hierarchical architecture of natural bone, subsequent work will implement gradient porosity designs that mimic the distinct pore gradients of cortical and cancellous bone. This approach was anticipated to markedly improve the load-bearing capacity of bionic scaffolds, enabling their adaptation to complex clinical scenarios requiring tailored mechanical performance.

### 3.3. Obvious Cellular Bridging Within PHS (55% Infill)

**Figure 3:**
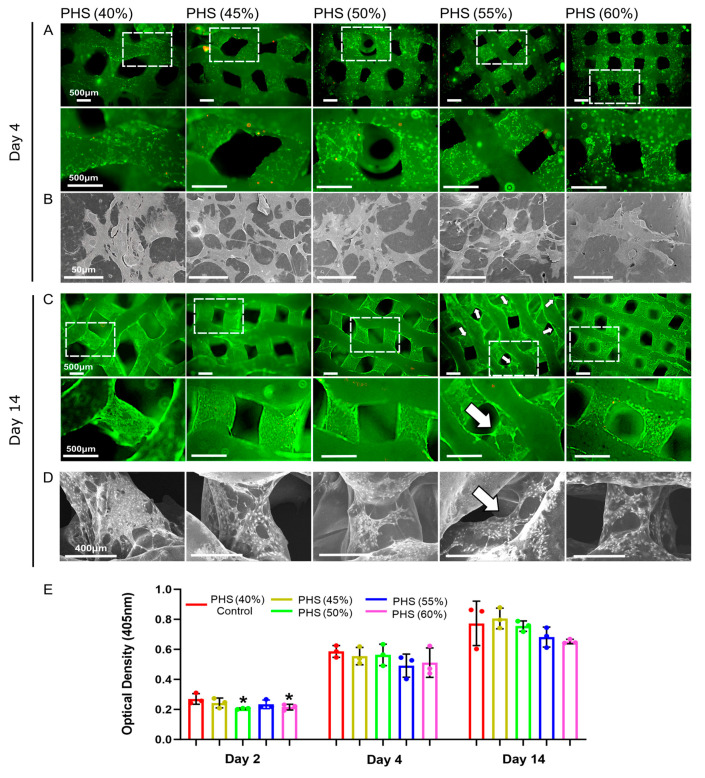
Analysis of bio-compatibility of bionic scaffolds. (**A**) Live/dead cell staining after 4 days of co-culture. (**B**) SEM image showing BMSC morphology after 4 days of co-culture. (**C**) Live/dead cell staining after 14 days of co-culture. (**D**) SEM image showing BMSC morphology after 14 days of co-culture. (**E**) CCK-8 test results after 2, 4, and 14 days of co-culture. The white arrow indicates that BMSCs exhibited more significant bridging within the pores of the 55% infill PHS. * = *p* < 0.05, the Student’s t-test and one-way analysis of variance (ANOVA) with post-hoc analysis to compare the difference between groups and the control group (40% infill PHS).

The live/dead cell staining and SEM results showed that, after 4 days of co-culture, BMSCs spread well and grew more pseudopods on the surface of PHS (45% infill), PHS (50% infill), and PHS (55% infill) compared to cells on PHS (40% infill) and PHS (60% infill) (Figure 3A,B). On day 14, all groups of the scaffolds showed more obvious cell proliferation. Notably, BMSCs showed more pronounced bridging within the pores of the PHS (55% infill) compared to the other four groups (Figure 3C,D). However, BMSCs on the surface of PHS (60% infill) scaffolds did not grow obvious pseudopods, which may be related to the poor circulation of oxygen, nutrients, and metabolic wastes because of the small pore size (376 ± 46 μm) [31]. Stretching alterations of the cytoskeleton will bring mechanical signaling stimuli to the cells, which may further activate the osteogenic pathway [47]. CCK8 results showed a noticeable increase in the cell viability after 2, 4, and 14 days of cell–scaffold co-culture (Figure 3E).

### 3.4. BMSCs with Optimal ALP Activity and Calcium Salt Deposition on PHS (55% Infill)

**Figure 4:**
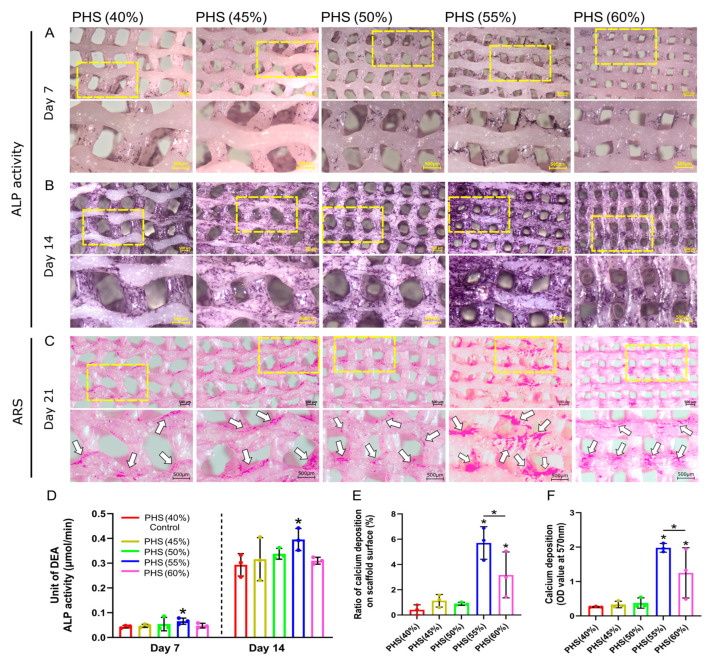
ALP activity of BMSCs and calcium salt deposition using Alizarin Red S (ARS) staining. (**A**) ALP staining on day 7. (**B**) ALP staining on day 14. (**C**) ARS results on day 21. White arrows represent calcium deposition on the scaffolds. (**D**) Quantitative analysis of ALP property after 7 and 14 days of co-culture for five scaffold groups (*n* = 3). (**E**) Ratio of calcium deposition on scaffold surface: the area of calcium salt deposition divided by the surface area of the scaffold. (**F**) Results of quantitative analysis of calcium deposition on day 21 (*n* = 3). * = *p* < 0.05, the Student’s t-test and one-way analysis of variance (ANOVA) with post-hoc analysis to compare the difference between groups and the control group (40% infill PHS). Additionally, calcium deposition also differs significantly between 55% and 60% infill-density scaffolds.

ALP is a significant enzyme generated by differentiated osteoblasts and is related to the bone matrix construction [48]. When the bionic scaffolds were co-cultured with BMSCs for 7 and 14 days, PHS (55% infill) showed the darkest ALP staining (Figure 4A,B). After 21 days of co-culture with the osteogenic medium, the ARS results showed that the PHS (55% infill) had the highest calcium salt deposition (Figure 4C). Notably, the regions exhibiting significant cellular bridging within the scaffold pores showed a significant amount of calcium salt deposition, especially at the lateral edges of the pores. The calcium salts mainly deposited on the surface of the remaining four groups’ scaffold (Figure 3C,D and Figure 4C). This phenomenon validated that cytoskeletal stretching may promote osteogenic differentiation of BMSCs. Moreover, calcium salts were deposited on the pore walls of PHS (55% infill) in a “concentric -circle-like” pattern, similar to the “Haversian system” of natural bone [49]. Cellular bridging created a continuous mineral network, mimicking natural bone’s self-reinforcing architecture. Calcium salts nucleated at BMSC-secreted collagen matrices, forming mineralized layers that integrate with the scaffold surface. The above concentric circle’s pattern conformed to scaffold pore geometry, enhancing interfacial bonding. However, future studies should incorporate shear resistance quantification and long-term stability testing to further evaluate the stability of calcium deposition patterns and mitigate delamination risks.

Further, the semi-quantitative analysis revealed that PHS (55% infill) exhibited the highest ALP activity, which was statistically different from the control group: PHS (40% infill), representing the most potent early osteogenic activity (Figure 4D). Further, the ALP activity of PHS (60% infill) was lower than that of PHS (55% infill), suggesting that the 55% infill density (corresponding to 465 ± 63 μm pore size) was the peak threshold for promoting early osteogenic differentiation of BMSCs. The semi-quantitative evaluation of ARS staining indicated that PHS (55% infill), possessed the highest calcium deposition ratio on the surface and optical density value, signifying the most pronounced calcium salt accumulation (Figure 4E,F).

BMSCs are highly mechanosensitive and can differentiate toward osteogenesis after sensing mechanical stimuli [50,51]. The stretching force brought to the cytoskeleton by cellular bridging is likely to activate endogenous osteogenic signaling pathways, such as the RhoA/ROCK2 signal pathway, Wnt pathway, bone morphogenetic protein (BMP) pathway, Notch pathway, mitogen-activated protein kinase (MAPK) pathway, and phosphatidylinositol 3-kinase (PI3K) and Protein Kinase B (PI3K/Akt) pathway [47,52]. Hence, bionic scaffolds with a 55% infill density that provide a higher degree of mechanical stimulation to cells are promising for osteogenesis applications and provide a reference for the design of artificial bone implants.

This study focused on optimizing scaffold parameters (e.g., infill density, mechanical properties, and osteogenic potential) through in vitro models. Our research also has limitations. Firstly, the internal pore structure of the bionic scaffolds was evaluated via SEM imaging. Future studies should incorporate micro-CT for comprehensive quantification of pore connectivity and spatial distribution. Secondly, current assessments prioritized compressive properties (e.g., modulus and yield strength). Future work will expand to tensile and shear performance evaluations under physiological loading conditions. Thirdly, bio-compatibility and osteogenic performance were validated using mouse bone marrow stromal cells (BMSCs). Future work will incorporate human-derived cells to validate result consistency and assess immunogenicity. Fourthly, static co-culture conditions may introduce nutrient diffusion gradients. We plan to implement dynamic, 3D scaffold-cell co-culture systems (e.g., bioreactors) to better mimic in vivo microenvironments. Fifthly, future work will conduct in vivo experiments to evaluate the risks of scaffold fragmentation or foreign body reactions, degradation kinetics, and bone and vascular regeneration efficacy based on long-term performance.

## 4. Conclusions

Our study demonstrates that FDM-printed PCL/HA gyroid scaffolds with a 55% infill density (pore size: 465 ± 63 μm) achieve a critical balance between hydrophilicity, mechanical strength, and osteogenic performance, mirroring the properties of native cancellous bone. The observed cellular bridging and calcium deposition patterns in PHS with the 55% infill indicate a bionic microenvironment that support osteogenesis through mechanotransduction. Looking forward, this work lays a foundation for three potential applications: (1) Digital technologies could enable patient-specific design and 3D printing of bone defect implants with gyroid structures at a 55% infill density, offering a promising approach to significantly reduce implant costs. (2) Combining our gyroid architecture with gradient infill strategies could mimic the hierarchical structure of cortical–cancellous bone interfaces, enabling load-bearing, site-specific defect repair. (3) Incorporating bioactive molecules (e.g., BMP-2 and VEGF) into the PCL/HA matrix may synergize with the observed mechanical stimulation effects to accelerate vascularized bone regeneration.

## Data Availability

The data of the study are all included in this study. Metadata are available upon reasonable request to the corresponding author.
